# The application of a dual-lead locking screw could enhance the reduction and fixation stability of the proximal humerus fractures: a biomechanical evaluation

**DOI:** 10.3389/fsurg.2024.1333670

**Published:** 2024-03-22

**Authors:** Eunju Lee, Hyeon Jang Jeong, Yeon Soo Lee, Joo Han Oh

**Affiliations:** ^1^ Department of BioMedical Engineering, School of BioMed Science, Daegu Catholic University, Gyoungbuk, Republic of Korea; ^2^ Department of Orthopaedic Surgery, Seoul National University College of Medicine, Seoul National University Bundang Hospital, Seoul, Republic of Korea

**Keywords:** proximal humerus, locked plating, dual-lead, lead ratio, compression, bone crush, insertion torque, stability

## Abstract

**Introduction:**

Bicortical screw fixation, which penetrates and fixes the near and far cortex of bone, has been conventionally used to achieve compressive fixation for fracture using screws. Open reduction and internal fixation using the locking plate are widely used for treating proximal humerus fractures. However, minimal contact between the bone and the locking plate can lead to an insufficient reduction. Theoretically, a dual-lead locking screw with different leads for the screw head and body could enhance the reduction and fixation stability of fragments in proximal humeral fractures without bicortical fixation, and achieve additional compression at the bone-plate-screw interface. This study assessed the insertion mechanics of the lead ratio of the dual-lead locking screw and its effect on the fixation stability of the proximal humerus fracture.

**Methods:**

A Multi-Fix® locking plating system composed of ∅ 3.5 mm locking screws and a locking plate was used to make a locked plating for Sawbone bone blocks and fourth-generation composite humeri. Two different types of Sawbone bone blocks were used to simulate the osteoporotic (10 PCF) and normal cancellous (20 PCF) bones. The lead of the screw head thread ($L_{\mathrm{head}}$) was 0.8 mm, and that of the screw body ($L_{\mathrm{body}}$) was 0.8, 1.25, 1.6, 2.0, and 2.4 mm, whose lead ratios ($R_{\mathrm{lead}}=L_{\mathrm{body}}/L_{\mathrm{head}}$) were 1.0, 1.56, 2.0, 2.5, and 3.0, respectively.

**Results:**

The dual-lead locking screw elevated the compression between the locking plate and the bone. The elevation in the compression due to the dual-lead thread became weaker for the cancellous bone when the lead of the screw body was more than twice that of the screw head. The plate/humerus compression with strong bone quality withstood higher dual-lead-driven compression.

**Discussion:**

A dual-lead locking screw of $L_{\mathrm{body}}=1.25\;\mathrm{mm}$ ($R_{\mathrm{lead}}=1.56$) is recommended for maximum rotational stability for the locked humerus plating. The screws with over $L_{\mathrm{body}}=1.6\;\mathrm{mm}$ ($R_{\mathrm{lead}}=2$) have no advantage in terms of the failure torque and maximum torsional deformation. Any locking dual-lead screw with a body thread lead of <1.6 mm ($R_{\mathrm{lead}}=2$) can be used without the risk of bone crush when surgeons require additional compression to the locked cancellous bone plating.

## 1 Introduction

The proximal humerus fracture is one of the major osteoporotic fractures among older adults (1–4). Aging could decrease bone mineral density (BMD) and increase osteoporosis risk. Osteoporosis does not provoke clinical symptoms; however, low BMD increases the risk of osteoporotic fracture and hinders the appropriate reduction and stable fixation during surgery (5).

Open reduction and internal fixation using the locking plate is one of the widely used methods for treating proximal humerus fractures (6). In a bone healing process, the periosteal vessels contribute to endochondral ossification. Locking the screw head and the plate hole inhibits normal and transverse movements between the surfaces of the bone and plate (7, 8). The restriction in the normal movement of the locked plating is advantageous because it exerts no or minimal compression on the periosteal vessels. However, locked plating does not achieve bone traction along the transcortical direction by screw fixation (9–13). In contrast, the use of bicortical fixation is limited in proximal humerus fractures because the proximal screws for the locked plating are directed to the articular cartilage. Therefore, obtaining accurate anatomical reduction through compressive fixation using a locking plate is a technically demanding procedure.

A dual-lead locking screw was designed to overcome this limitation. Theoretically, the dual-lead screw could achieve additional reduction through the limited compression originating from the different lead ratios. Therefore, this study aimed to experimentally assess the insertion mechanics of the dual-lead locking screw and its effect on the fixation stability of proximal humerus fractures. Eventually, this study will provide surgeons with the biomechanical clue for the effective utilization of dual-lead locking screws when they want to apply additional anatomical reduction at performing a locked plating for the proximal humerus fracture.

## 2 Materials and methods

### 2.1 Test design

**Figure 1:**
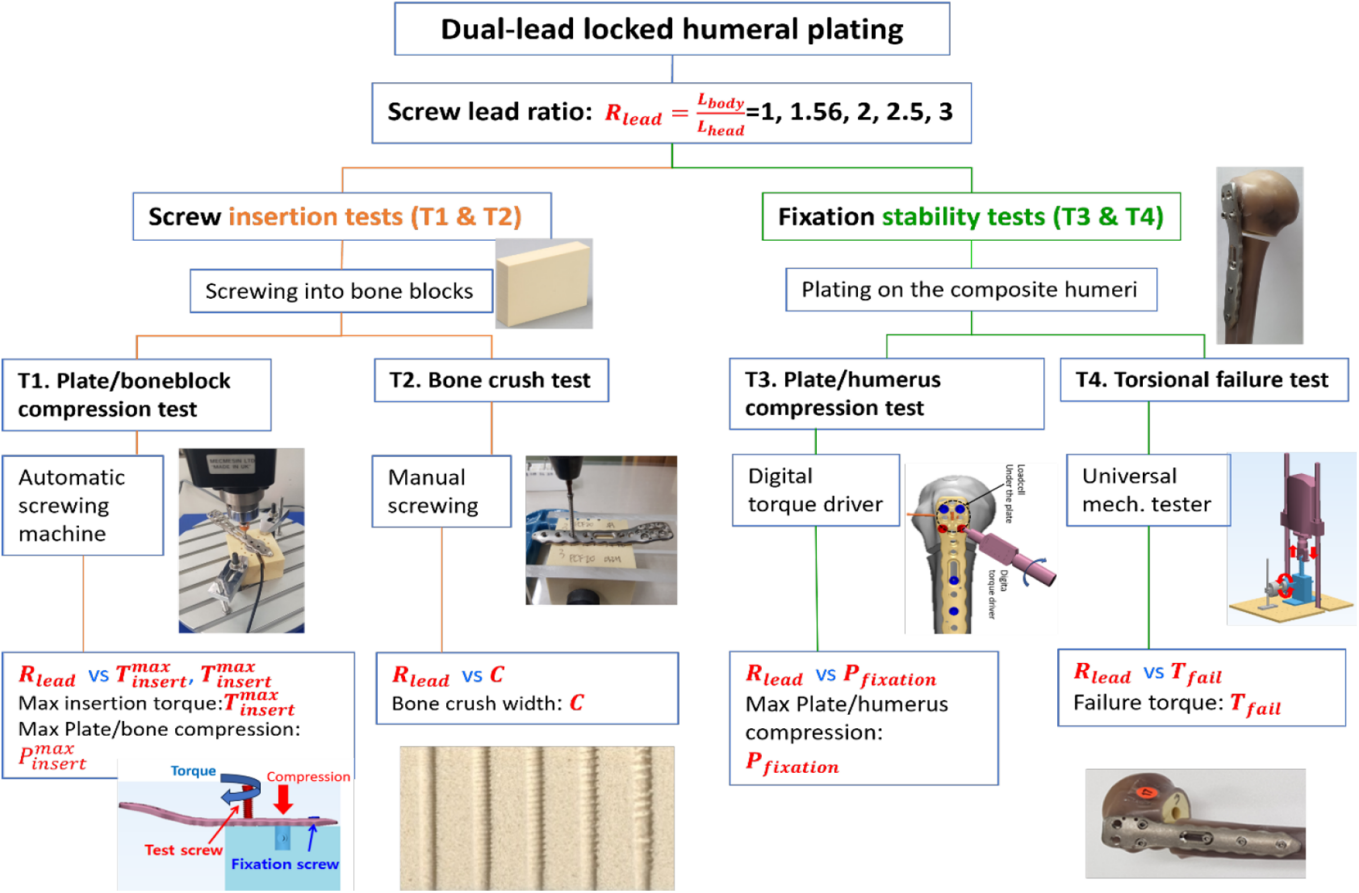
Scheme of tests. Four kinds of tests (T1–T4) were designed to assess the effect of the lead ratio on screw insertion mechanics and failure properties.

The experiments were designed in two main testing modes: screw insertion and fixation stability tests (Figure 1). The screw insertion tests assessed the intrinsic screwing features of the locking head screws, whereas the fixation stability tests evaluated the structural stability of the locked proximal humerus plating complex.

### 2.2 Ethics approval and consent to participate

This study does not include any data from individuals.

### 2.3 Materials

#### 2.3.1 Screws and plates for the fixation of proximal humerus fractures

The tested fixation system for the proximal humerus fracture was a Multi-Fix® locking plating system (ORTHOTECH Co., Ltd, Republic of Korea). It is composed of a locking head and locking head screws that are made of titanium.

**Figure 2:**
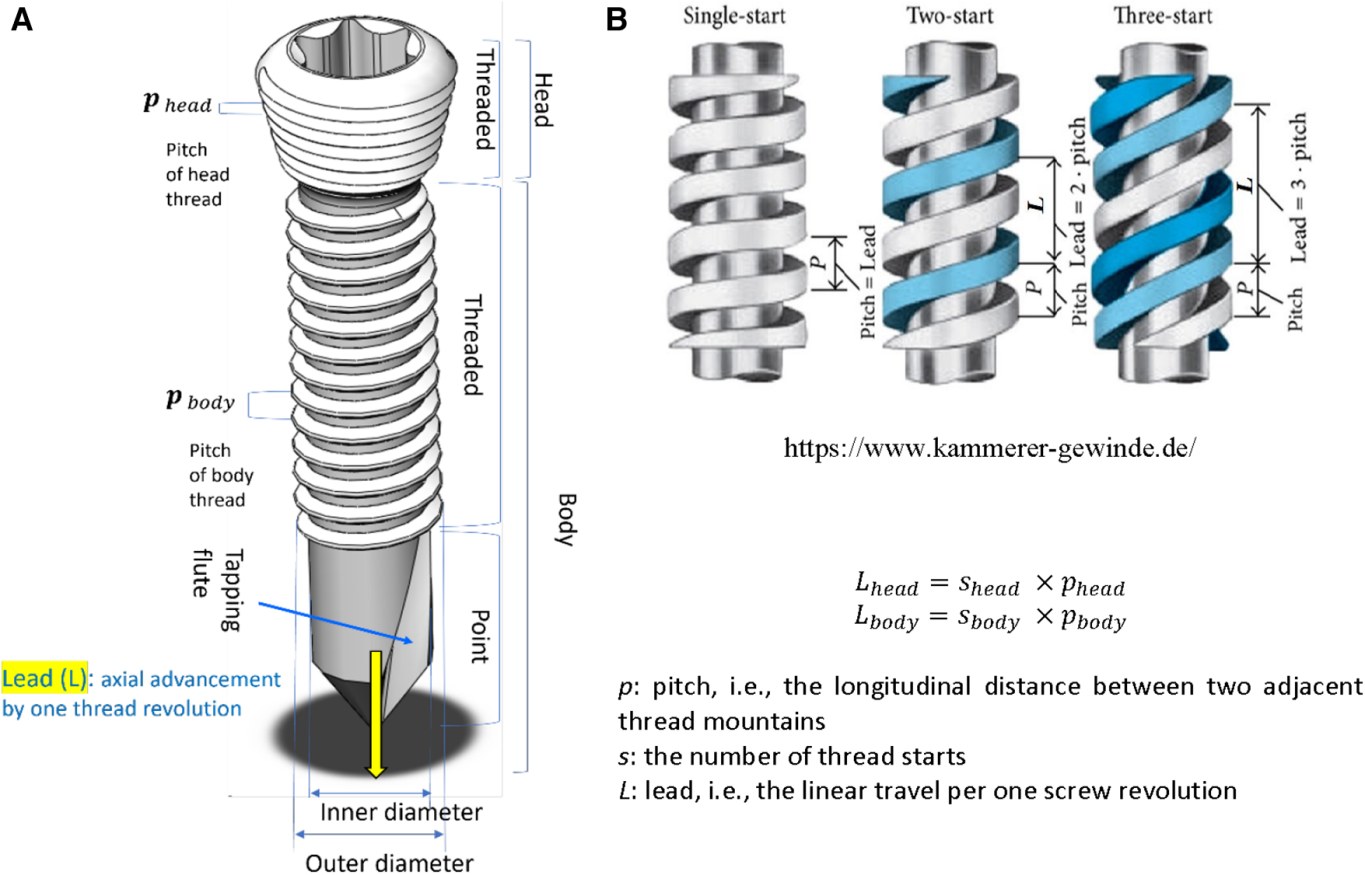
Screw structure. (**A**) The anatomy of a locking head screw, (**B**) Single-start and multi-start threads $\begin{array}{c} L_{\mathrm{head}}=s_{\mathrm{head}}\;\times p_{\mathrm{head}}\\ L_{\mathrm{body}}=s_{\mathrm{body}}\;\times p_{\mathrm{body}} \end{array}$. *p*: pitch, i.e., the longitudinal distance between two adjacent thread mountains. *s*: the number of thread starts. *L*: lead, i.e., the linear travel per one screw revolution.

In screw mechanics, a lead is the linear travel per screw revolution, while a pitch is the distance between the crests of adjacent threads*.* The number of thread starts is the number of neighboring threads that simultaneously start screwing*.* Therefore, a lead is the multiplication of a pitch and the number of thread starts (Figure 2).

The head and body threads of all screws are single-start, with ∅ 3.5 mm of nominal screw diameter. The lead of the head thread ($L_{\mathrm{head}}$) was 0.8 mm and the leads of the body ($L_{\mathrm{body}}$) were 0.8 mm, 1.25 mm, 1.6 mm, 2.0 mm, and 2.4 mm. As given in Equation 1, the lead ratio ($R_{\mathrm{lead}}$) of a locking screw was defined as the ratio of the thread lead of the screw body with respect to that of the screw head,

(1) $$R_{\mathrm{lead}}=L_{\mathrm{body}}/L_{\mathrm{head}}$$

All the screws of $R_{\mathrm{lead}}\neq 1$ are dual-lead screws. The screw having the same 0.8 mm lead for its head and body is a single-lead screw ($R_{\mathrm{lead}}=1$). In contrast, other screws with different leads for their head and body are $R_{\mathrm{lead}}>1$ and are referred to as “dual-lead screws” (Table 1). Consequently, one single-lead and four dual-lead screws were compared. Simplified plates with seven holes were made since the fixation stability test of the plated humerus uses a maximum of seven screws (Table 2).

**Table 1:** Locking head screws.

Model (Manufacturer)	Multi-Fix® locking screw (ORTHOTECH Co., Ltd, Rep. Korea)						
Test usage	Anchor		Test screws				
Picture	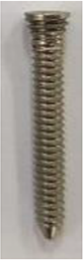	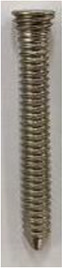	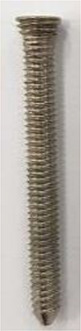	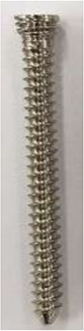	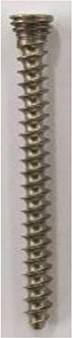	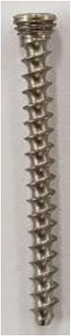	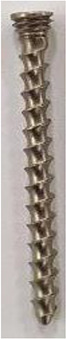
Material	Grade V titanium alloy (Ti-6Al-4V-ELI)						
Nominal diameter (mm)	∅3.5 (body)						
Starts of thread	Single-start (for all head and body threads)						
$L_{\mathrm{head}}=p_{\mathrm{head}}$ (mm)	0.8						
$L_{\mathrm{body}}=p_{\mathrm{body}}$ (mm)	0.8			1.25	1.6	2.0	2.4
Lead Ratio
	1			1.56	2	2.5	3
$R_{\mathrm{lead}}=L_{\mathrm{body}}/L_{\mathrm{head}}$	Single-lead screw			Dual-lead screw			
Length (mm)	26	28	40				

**Table 2:** Locking humerus plates.

Model (Manufacturer)	Multi-Fix® proximal humerus locking plate (ORTHOTECH Co., Ltd, Rep. Korea)	
Test usage	Screw insertion test	Fixation stability test
Hole thread	Single-start, 15 holes	Single-start, 7 holes
Material	Grade II Commercially pure titanium	
Picture	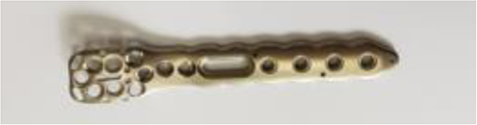	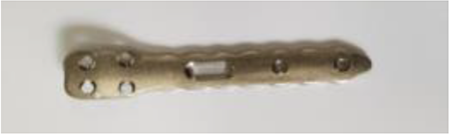

#### 2.3.2 Artificial bone blocks and composite humeri

Synthetic bone blocks and humeri were used for the screw insertion tests (plate/bone compression test [T1] and the bone crush test [T2]) (Table 3). The densities of the bone blocks (Sawbones, SKU1522-01, USA) were 10 and 20 pounds per cubic foot (PCF), corresponding to osteoporotic and normal cancellous bones, respectively (14, 15). The artificial humerus was a fourth-generation composite humerus (Sawbones, SKU3404, USA) with a mean density of 17 PCF (16).

**Table 3:** Artificial bones (data from available at: https://www.matweb.com).

Manufacturer		Sawbones		
Manufacturing country		United States of America		
		Bone block		Fourth-generation composite humerus (HS4, Model 3404, normal density)
Density	PCF	10 (osteoporotic cancellous)	20 (normal cancellous)	Cortical 100 Cancellous 17
	g/cm^3^	0.16	0.32	Cortical 1.64 Cancellous 0.27
Poissons ratio		0.3	0.3	0.3
Compressive strength (MPa)	Strength	2.2	8.4	Cortical 157 Cancellous 6
	Modulus	58	210	Cortical 16700 Cancellous 155
Picture		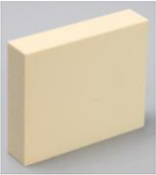		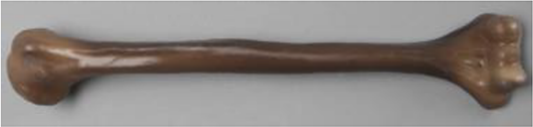

### 2.4 Screw insertion tests (plate/bone compression [T1] and the bone crush tests [T2])

The screw insertion tests aim to assess interactive mechanical responses between the locking head screws and the bone block. As in Figure 2, the screw insertion tests can be subdivided into the plate/bone compression test (T1) and the bone crush test (T2).

#### 2.4.1 Plate/bone block compression test (T1)

**Figure 3:**
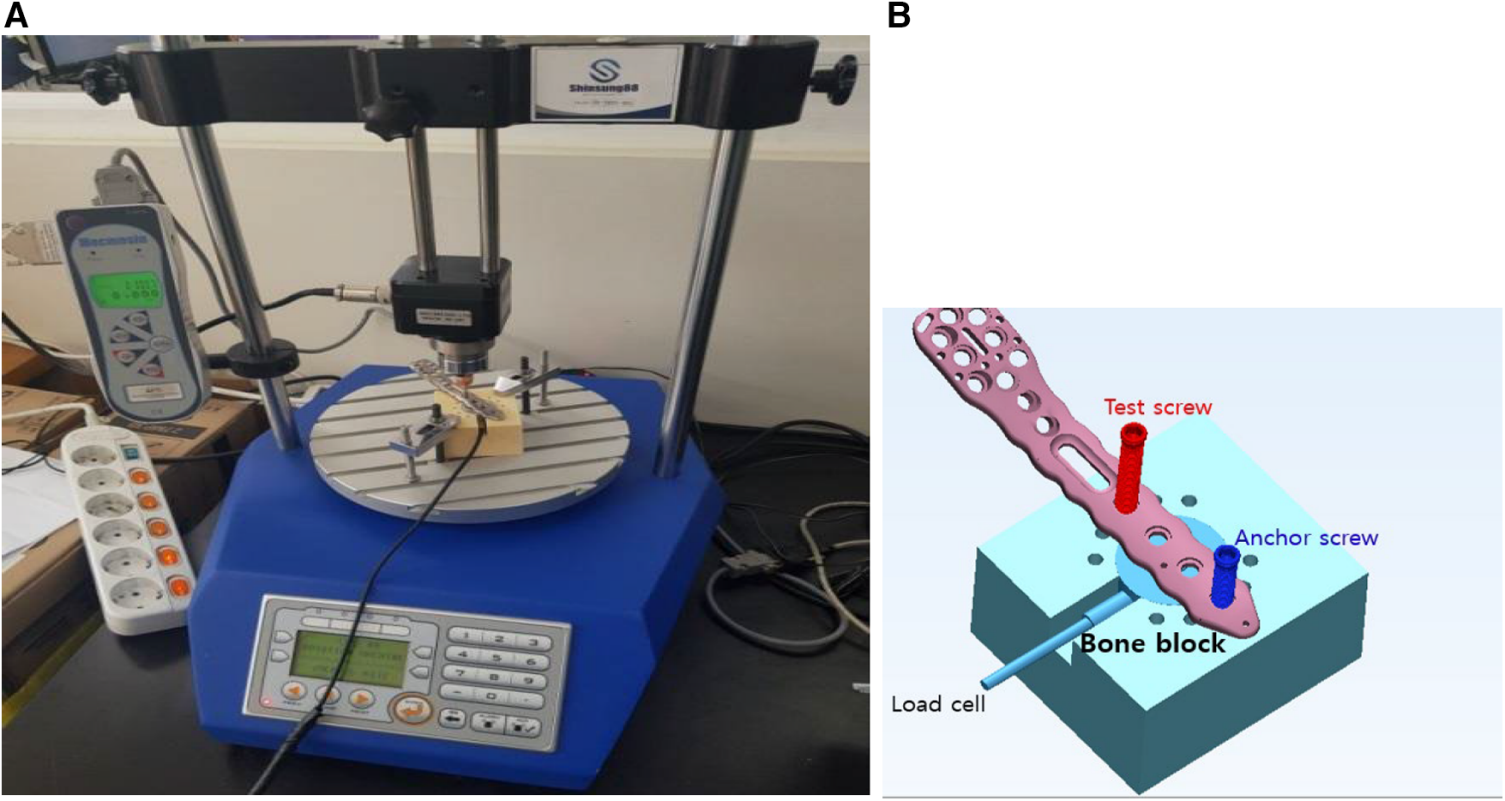
Testing setup for the plate/bone compression test. (**A**) An automatic screw insertion tester, (**B**) the loadcell settles in a cylindrical housing in a bone block and can measure the compression applied by the plate's contact against the bone block.

The plate/boneblock compression test (T1) is an angular displacement control test. As the screw enters the bone block, the computer records the insertion torque and the plate's compression force against the bone block. An automatic screw insertion tester (Shinsung 88, SS-5511, Shinsung Co., Republic of Korea) was newly developed based on Mecmesin torque testing parts and software (Mecmesin, a part of PPT Group UK Ltd, United Kingdom) (Figure 3A). To measure the compressive force of the plate against the bone block, a load cell (UNCDW-500N, Unipulse Co., Japan) was settled on the cylindrical housing in the bone block. The compression exerted by the plate was measured as the test locking screw head entered the locking plate's screw hole (Figure 3B).

The holes and housing for the load cell were precisely machined for repeatable tests. The holes were machined to ∅ 2.8 mm in diameter and 35 mm in length, serving as pilot holes for the ∅ 3.5 mm screw. A 28 mm long screw was inserted as a stabilization anchor, and subsequently, 40 mm long screws were tested. Based on ASTM F543A2, the automatic screw tester rotated screws at 3 rpm with an axial compression of 1.1 kg*_f_*. Real-time load cell and torque data were recorded on the computer.

#### 2.4.2 Bone crush test (T2)

Dual-lead locking screw plating will make a compression between the plate and the bone, and the bones contacting the screw's body can be crushed due to the compression. If the crush is enormous, bone healing to fill the crushed space will be prolonged. Therefore, understanding the extent of bone crush emanating from thread compression is crucial.

**Figure 4:**
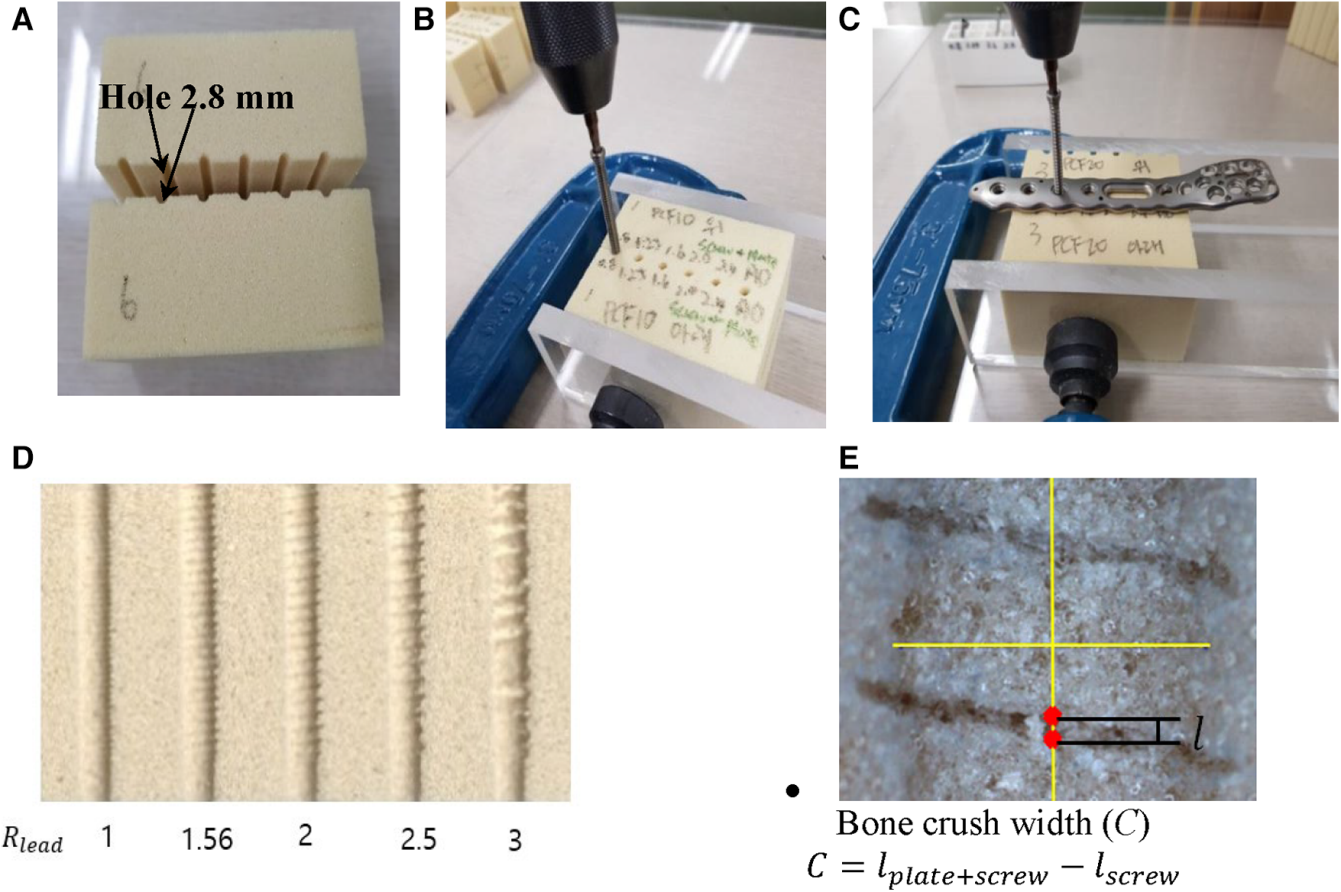
The measurement process of crushed bone length. (**A**) Two bone blocks with half pilot holes on its wall, (**B**) the screw insertion into the pilot holes formed by clamping corresponding blocks, (**C**) the screw insertion through the locking plate and then into the pilot holes formed by clamping corresponding blocks, (**D**) footprint formed by dual-lead locking head screws of various lead ratios, and (**E**) the calculation of the bone crush width.

Here, 10 PCF and 20 PCF bone blocks were used (Table 3). Figure 4 depicts the measurement process of the crushed bone's width. First, two bone blocks were precisely machined into halves of pilot holes on one of the side walls (Figure 4A). Thereafter, two blocks were clamped such that the halves of their pilot holes matched perfectly constructed holes. The footprint created by only the screw insertion was excluded in observing the bone crush due to the dual-lead locked plating. Therefore, the footprints without the plate and the dual-lead locked plating were separately measured (Figures 4B,C, respectively). The screw insertion without the plate was performed while the insertion torque remained <0.1 N-m. Conversely, the screw insertion with the plate was manually performed until the screw/plate locking was complete because the automatic screw tester lacked sufficient space for the bone block and metal clampers. Figure 4D displays the footprint created by dual-lead locked plating of various lead ratios.

The footprints were observed in detail using the NiKon Tool Measuring Microscope MM-800/l (NiKon Co., Japan). The microscope images were imported to CAD software (SolidWorks Co., Dassault Systèmes, France), and the footprint created by the screw thread was measured using the scale bar (Figure 4E). Moreover, the thread footprint can vary depending on the location; therefore, the footprint at the center of the whole screw footprint area was measured. The footprint width (*l*) was defined as the longitudinal length of the central thread footprint.

Any screw thread traveling through the bone will leave its footprint. The true bone crush emanating from dual-lead locked plating should eliminate the footprint created by the plating-free invasion of the screw body thread. Consequently, the bone crush width (*C*) was calculated as the width remaining after subtracting the footprint width of the screw insertion ($l_{\mathrm{screw}}$) from the footprint width of dual-lead locked plating ($l_{\mathrm{plate}+\mathrm{screw}}$) as illustrated below:

(2) $$C=l_{\mathrm{plate}+\mathrm{screw}}-l_{\mathrm{screw}}$$

where $l_{\mathrm{screw}}$ is the thread footprint width formed by screw insertion into a bone block without plating, and $l_{\mathrm{plate}+\mathrm{screw}}$ is the thread footprint width formed by screw insertion through the locking plate and a bone block.

### 2.5 Fixation stability tests (plate/humerus compression [T3] and torsional failure load [T4] tests)

The structural stability of the proximal humerus fracture fixed with a locked plating was evaluated by plate/humerus compression test (T3) and torsional failure load test (T4). Unlike the screw insertion tests (T1 and T2), these fixation stability tests use the composite humeri (Table 2) to evaluate the locked proximal plating fixation considering anatomical topology and structural deformity.

#### 2.5.1 Plate/humerus compression test (T3)

**Figure 5:**
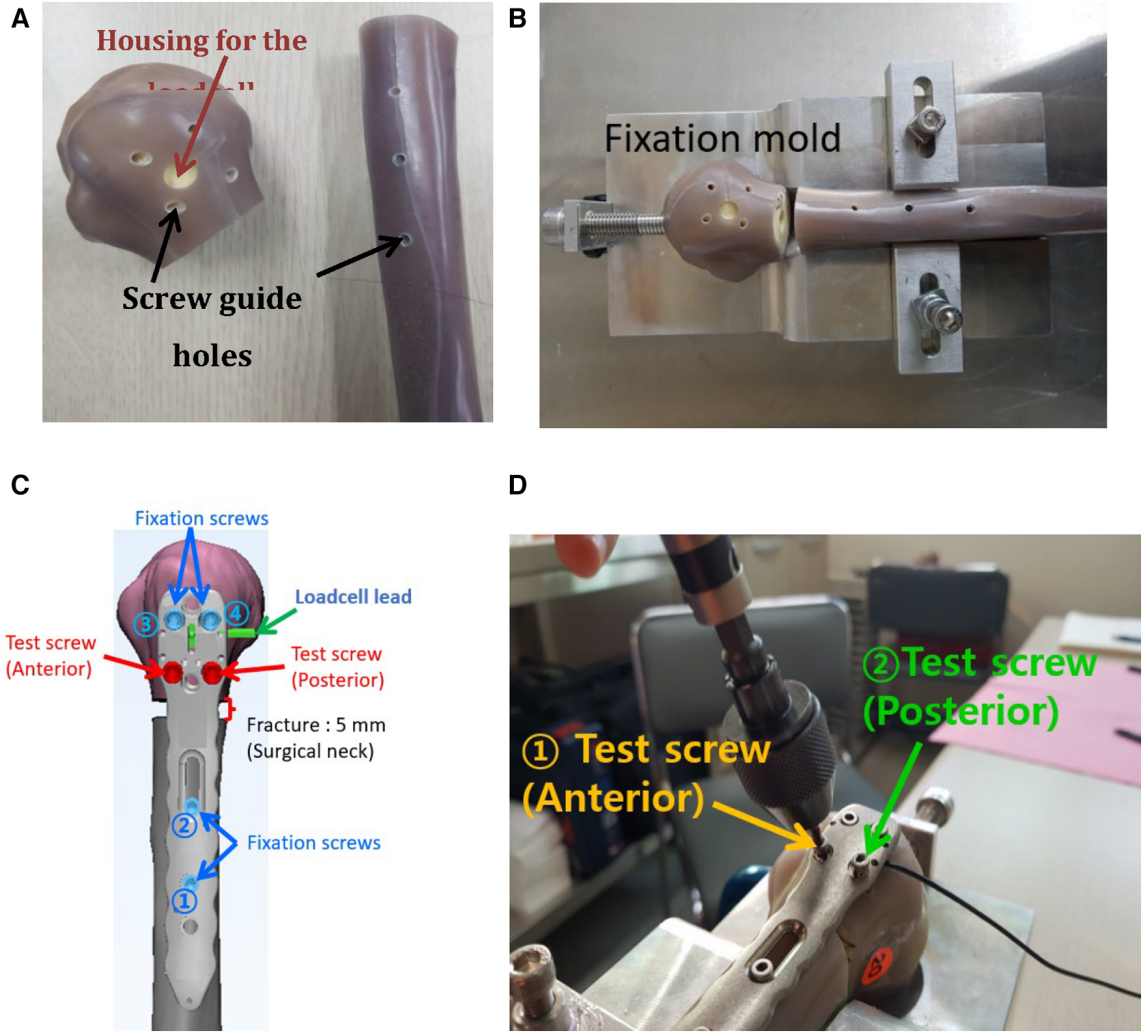
The process of the plate/humerus compression test. (**A**) Screw pilot holes and the loadcell housing were precisely machined, (**B**) the humerus was mounted on the fixation mold for repeatable humerus alignment, (**C**) a scheme representing the locations and installation sequence of fixation screws and a simulated fracture, and (**D**) real testing scene.

The composite humerus was precisely machined to simulate a proximal humeral fracture of a 5 mm gap (17). Furthermore, the screw pilot holes and load cell housing were machined (Figure 5A). A fixation mold was machined using a 5-axis computer numerical control machine to rigidly hold the humerus to mimic the medial anatomical contour of the proximal humerus. Thus, once the humerus fracture compartments have been fixed on the mold, they typically maintain the alignment of the previous specimens (Figure 5B). Figure 5C illustrates that distal and proximal fixation screws were inserted sequentially, keeping the loadcell subjected to a compression <25 N. We chose the 25 N compression at which fixation screws could complete locked plating and the plate's under surfaces engaged with the fixation screws could make contact with the humerus. Finally, the plate/humerus compression was measured sequentially at every locking of the anterior and posterior test screws completed (Figure 5D).

#### 2.5.2 Torsional failure test (T4)

**Figure 6:**
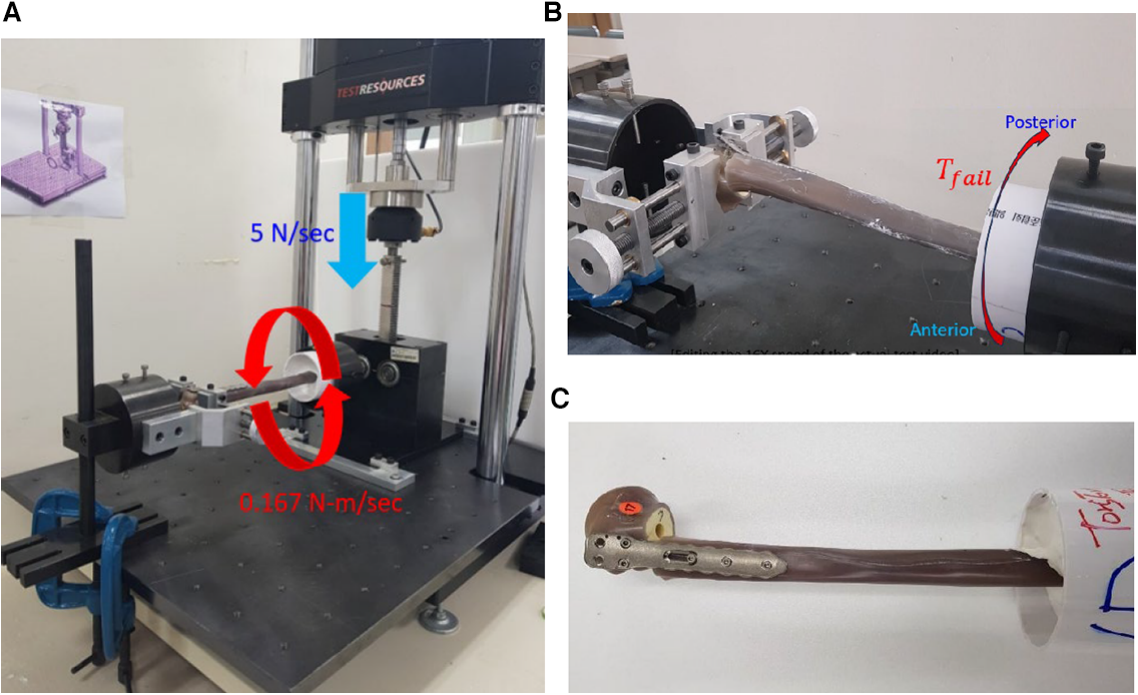
The fixation torsional stability test. (**A**) The mechanical tester, customized rack and pinion system enable the converting of the linear movement into an axial torsion. The force rate of 5 N/s was a converted torque rate of 0.167 N-m/sec, (**B**) the humerus fixation failed at the maximum internal torque, and (**C**) the failed specimen due to the torsion.

The torsional stability of the locked proximal humerus plating was evaluated by measuring failure torque. The specimens used for T3 were utilized for this test. The loadcells from the specimens used for T3 were removed by releasing the four proximal screws and re-inserting them until the screw heads were completely locked. The specimen's distal end was fixed in the plastic pipe using Kirschner wires and plastered to be clamped with the rotational metal cylinder (Figure 6). The dynamic tester 810E (Testresources Co., USA) was used for the mechanical test. A customized rotational jig converted the vertical linear movement of the dynamic tester into a torsional movement of the metal cylinder clamping the distal end (Figures 6A,B). The loading head of the dynamic tester moved at a downward rate of 5 N/s, corresponding to a torsion rate of 0.167 N-m/s (18, 19). Due to the torque, the distal humerus rotates externally while the humeral head is fixed at the end of the rotation jig.

## 3 Results

### 3.1 Plate/bone block compression test (T1)

**Figure 7:**
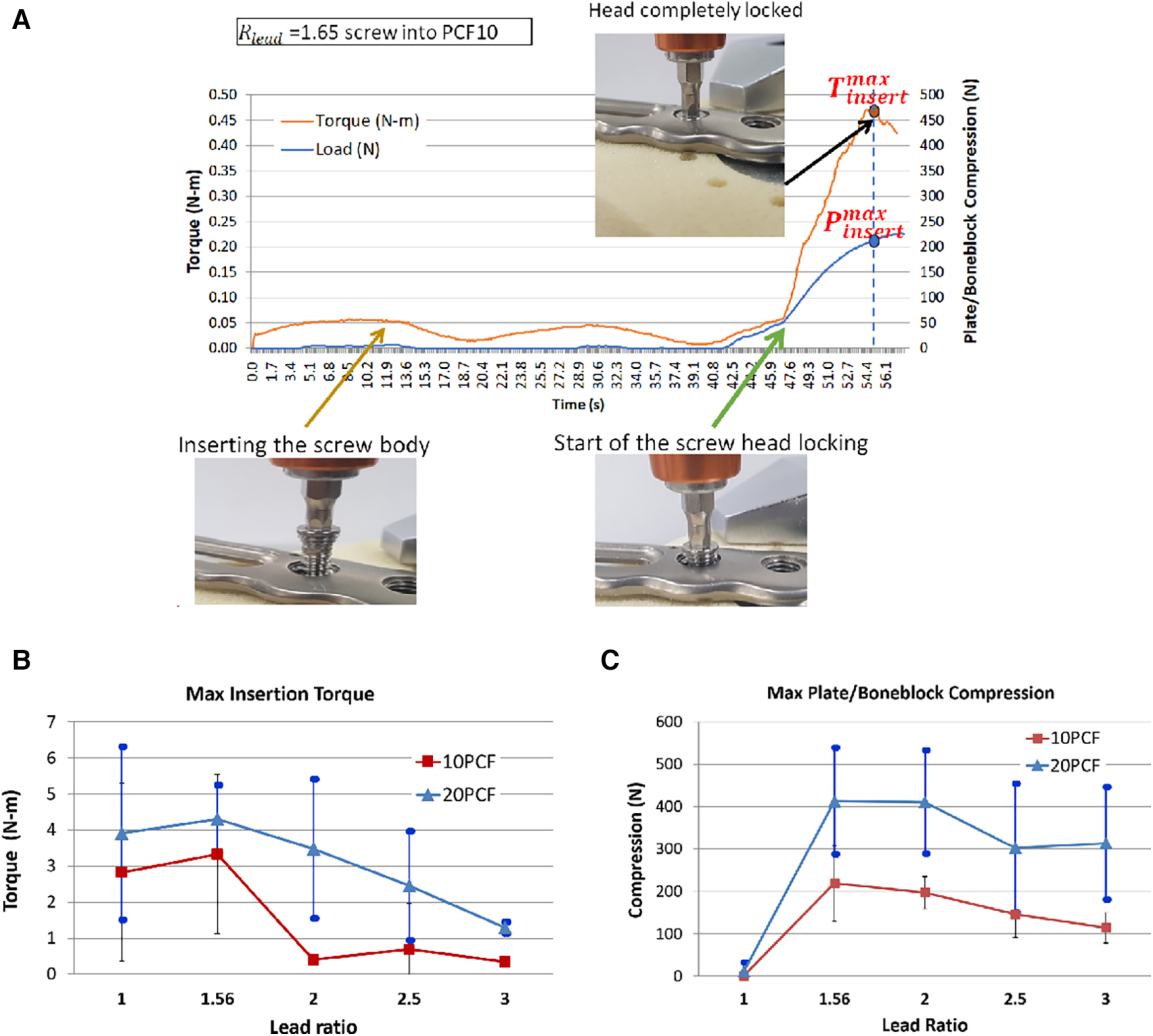
The plate/boneblock compression test results. For each condition, 10–12 successful test results were obtained: (**A**) The real-time record of mechanical response according to the screw insertion process of a locked plating with the bone block. As the automatic screw tester began to rotate the ∅ 1.25 mm screw (lead ratio; 1.65 = 1.25/0.8) diameter, the screw body entered the pilot hole, and the insertion torque slightly increased because the self-tapping flutes engaged the pilot hole entrance, (**B**) The maximum insertion torque measured at the complete locked plating, (**C**) The maximum plate/bone block compression.

Figure 7A reveals a locked plating with the bone block and the mechanical response according to the screw insertion process. As the automatic screw tester began to rotate the ∅ 1.25 mm screw (lead ratio; 1.65 = 1.25/0.8) diameter, the screw body entered the pilot hole, and the insertion torque slightly increased because the self-tapping flutes engaged the pilot hole entrance. The insertion torque and plate/bone compression remained <0.5 N-m and 0 N, respectively, until the screw head met the locking plate. As long as only the screw body was inserted into the bone block, the insertion torque did not increase since the screw needed to construct a thread passage on the wall of the pilot hole rather than drilling the opposing bone volume. Immediately the screw head contacted the plate, the screw-head and plate locking started, and the inertion torque and plate/bone compression increased rapidly. When the plate/screw locking was completed, the insertion torque reached its maximum. At this maximum torque, the value of the plate/bone compression was defined as the maximum compression, as it marginally increased and slightly decreased again to the level at the maximum torque.

Seeing the maximum insertion torque, only $R_{\mathrm{lead}}=1.56$ cases had a higher value than the single lead screw ($R_{\mathrm{lead}}=1$). Other screws of $R_{\mathrm{lead}}>1.56$ demonstrated lower insertion torque than the single lead screw (Figure 7B). At the 10 PCF bone block, the maximum insertion torques were 2.8 ± 2.5, 3.3 ± 2.2, 0.4 ± 0.1, 0.7 ± 1.3, and 0.3 ± 0.1 N-m for $R_{\mathrm{lead}}=1.0,\;1.56,\;2.0,\;2.5,\;\mathrm{and}\;3.0$, respectively. At the 20 PCF bone block, the maximum torques were 3.9 ± 2.4, 4.3 ± 0.9, 3.5 ± 1.9, 2.5 ± 1.5, and 1.29 ± 0.2 N-m for $R_{\mathrm{lead}}=1$, 1.56,2,2.5,and3, respectively. Typically, the highest value was observed for $R_{\mathrm{lead}}=1.56$ at 10 and 20 PCF and other dual-lead screws revealed lower values than the single lead screw.

As the locking mechanism works, the locked plating induces plate/bone block compression due to the dual-lead structure (Figure 7C). At the 10 PCF bone block, the mean maximum plate/bone block compressions were 1.8 ± 2.7, 218.9 ± 89.6, 197.1 ± 38.2, 146.1 ± 36.1 N, and 114.4 ± 36.1 N for $R_{\mathrm{lead}}=1.0,\;1.56,\;2.0$, 2.5,and3.0, respectively. The highest compression was observed for $R_{\mathrm{lead}}=1.56$, and it decreased to 50% of that of $R_{\mathrm{lead}}=1.56$ with increased $R_{\mathrm{lead}}$. At the 20 PCF bone block, the mean maximum plate/bone block compressions were 11.1 ± 21.9, 413.3 ± 125.9, 410.9 ± 122.3, 302.8 ± 152.2, and 313.8 ± 133.2 N for $R_{\mathrm{lead}}=1.0,\;1.56,\;2.0,\;2.5,\;\mathrm{and}\;3.0$, respectively. Similar to 10 PCF, the highest value was observed for $R_{\mathrm{lead}}=1.56$; however, it was very close to that for $R_{\mathrm{lead}}=2$. As $R_{\mathrm{lead}}$ rose to 2 and 2.5, the compression decreased by approximately 25% of that of $R_{\mathrm{lead}}=1.56$. The standard deviation of 20 PCF cases was larger than that of 10 PCF cases.

### 3.2 Bone crush test (T2)

**Figure 8:**
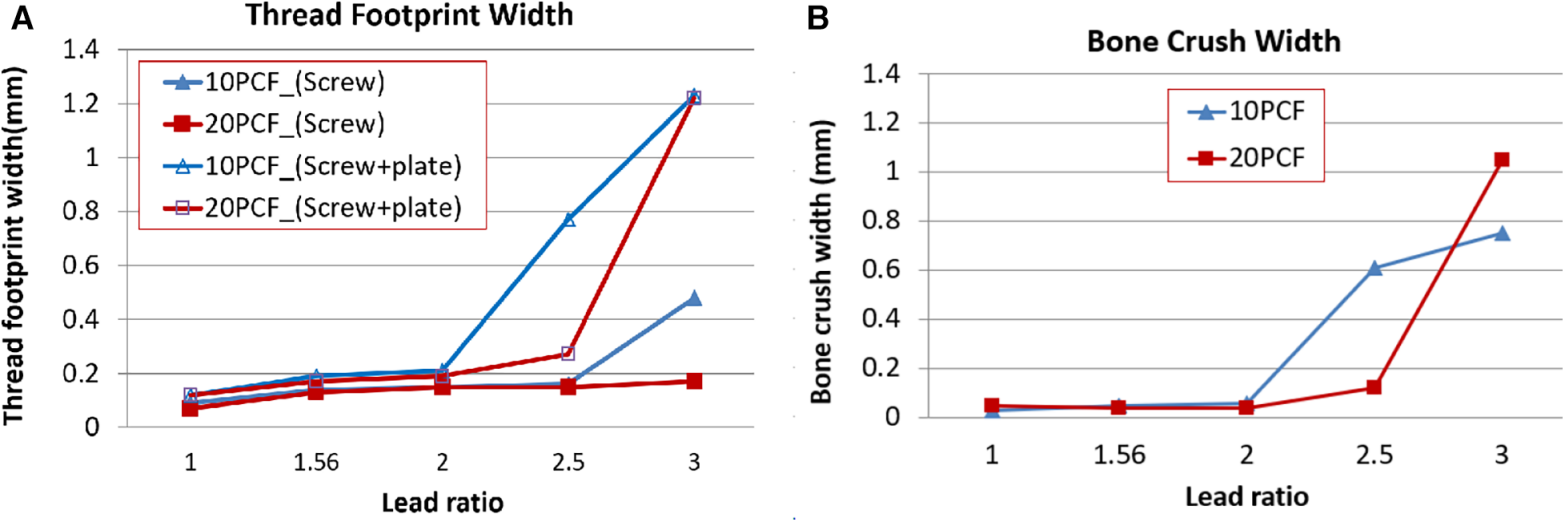
The bone crush test results. For each condition, a single successful test result was collected. (**A**) Thread footprint widths made using only the screw insertion and locking screw/plate plating, respectively. (**B**) The bone crush width calculated using Equation 2.

The footprint width due to the screw insertion without the plate was <0.2 mm, except for the insertion of $R_{\mathrm{lead}}=3.0$ screw into the 10 PCF bone block (Figure 8A). This exception might result from the abrupt large stepping of the screw body thread of $R_{\mathrm{lead}}=3$ into the osteoporotic bone. The footprint width of the locked plating increased significantly for the screws over $R_{\mathrm{lead}}=2.5$. The increase was more prominent in the locked plating of $R_{\mathrm{lead}}=3$.

The bone crush due to locked plating was not large for the cases of $R_{\mathrm{lead}}=1.0,\;1.56,\;\mathrm{or}\;2.0$ (Figure 8B). However, it increased with $R_{\mathrm{lead}}>2$, which was more prominent in the cases of $R_{\mathrm{lead}}=2.5\;\mathrm{and}\;3$ and $R_{\mathrm{lead}}=3$ for 10 and 20 PCF, respectively.

### 3.3 Plate/humerus compression test (T3)

Locked plating to the proximal humerus induces plate/humerus compression. At the completion of the locked plating, the plate/humerus compression was measured by the compression sensor installed under the plate. The plate/humerus test was performed only for $R_{\mathrm{lead}}=1.0,\;1.56,\;2.0,\;\mathrm{and}\;2.5$ cases because the plate/bone block compression did not differ for $R_{\mathrm{lead}}=3$ cases compared with $R_{\mathrm{lead}}=2.5$ cases and the bone crush width was too large for $R_{\mathrm{lead}}=3.0$.

**Figure 9:**
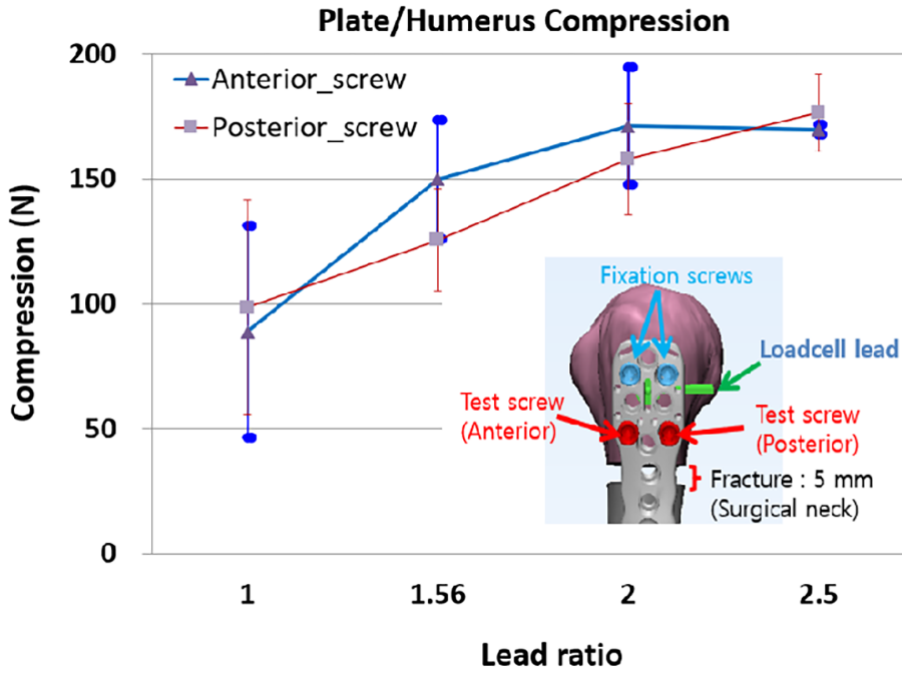
The plate/humerus compression, due to the sequential locked plating of anterior and posterior screws. For each condition, six humerus specimens were tested.

The insertion of a $R_{\mathrm{lead}}=1.0$ test screw induced the compression of 39–138 N (88.93 ± 43) higher than the values observed during the plate/bone block insertion tests because the fixation screws were inserted before the test screws (Figure 9). The anterior test screw demonstrated an increase in the compression for $R_{\mathrm{lead}}=1.56\;\mathrm{and}\;2.0$, and almost no change was observed for $R_{\mathrm{lead}}=2.5$. The posterior test screw compression increased with $R_{\mathrm{lead}}$.

### 3.4 Torsional failure test (T4)

At the point where an abrupt decrease in torque, the failure torque and rotation were identified. We concluded that the locked plating with $R_{\mathrm{lead}}=2.5$ screw is not useful because the plate/humerus compression for $R_{\mathrm{lead}}=2.5$ slightly changed from $R_{\mathrm{lead}}=2.0$ and the bone crush was immense. Therefore, this torsional failure test was performed only for $R_{\mathrm{lead}}=1,\;1.56$, and 2.0.

**Figure 10:**
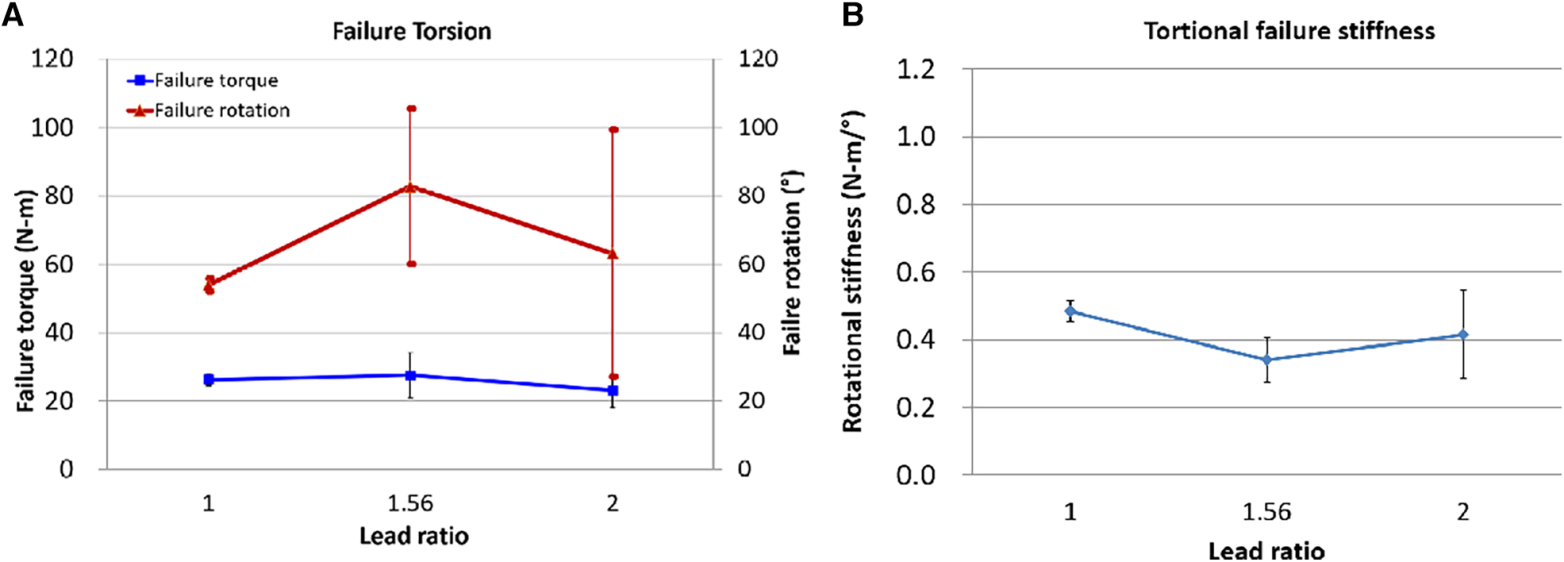
The fixation torsional failure test results. (**A**) Failure torque and rotation, (**B**) Torsional failure stiffness = (failure torque)/(failure rotation).

For each $R_{\mathrm{lead}}$, the six specimens used for the plate/humerus compression test were utilized for this torsional failure test. The specimens showing bone breaks outside the plating or breaks of the screw at low torsion were not considered: one specimen of $R_{\mathrm{lead}}=1.0$ and two of $R_{\mathrm{lead}}=2.0$. Consequently, results from the first successful four specimens each of $R_{\mathrm{lead}}=1.0,\;1.56,\;\mathrm{and}\;2.0$ were collected. The mean maximum failure torque was 26.2 ± 1.8, 27.5 ± 6.6, and 23.0 ± 4.9 N-m for $R_{\mathrm{lead}}=1.0,\;1.56,\;\mathrm{and}\;2.0$, respectively. The failure rotation for $R_{\mathrm{lead}}=1.56$ was 82.8° ± 22.2° which was 52% and 30% higher relative to those of $R_{\mathrm{lead}}=1.0\;\mathrm{and}\;2.0$, respectively (Figure 10A). The failure rotation for $R_{\mathrm{lead}}=1.56$ was 82.8° ± 22.2° which was 52% and 30% higher relative to those of $R_{\mathrm{lead}}=1\;\mathrm{and}\;2$, respectively. Torsional stiffness was lower for $R_{\mathrm{lead}}>1$ and lowest at $R_{\mathrm{lead}}=1.56$ (Figure 10B).

## 4 Discussion

This study assessed the biomechanics of the dual-lead locking screw and the fixation stability for various screw lead ratios. During the screw insertion tests (T1 and T2), we observed plate/bone block interactive mechanics when the dual-lead screws were inserted into locking plates and bone blocks. Furthermore, the fixation stability tests of the locked plating of the proximal humerus fracture (T3 and T4) demonstrated the effect of the dual-lead screws.

Accurate reduction and firm fixation are critical to healing after a fracture. However, achieving a firm fixation in elderly patients with osteoporosis remains challenging (4, 20–22). Several kinds of screw compression mechanisms have been utilized for sufficient anatomical reduction and stable fixation of fractured fragments (23). Combinations of different diameters, lagging lengths, or leads create various interfragmentary screw compression. An increasing thread diameter can induce interfragmentary compression because the larger proximal diameter struggles to advance into the narrower distal screw diameter. Stryker TwinFix (Stryker, Kalamazoo, MI, USA) has identical thread pitch and lead but different proximal and distal diameters. In addition, a longer lagging length (i.e., non-threaded length) induces more interfragmentary compression since the compressed bone length is proportional to the lagging length. A varying screw lead design can draw a gradual compression between fracture fragments as the screw advances. Acumed Acutrak 2 Mini (Acumed, Hillsboro, OR, USA) used a continuously varying lead screw through the whole thread. In contrast, KLS Martin HBS 2 Midi (KLS Martin, Tuttlingen, Germany) adopted the dual-lead screw whose proximal and distal leads differ. The dual-lead screw is a screw with different head and body leads. Owing to the different stride lengths due to different proximal and distal leads, a compression exists between the plate and bone fragments as the head's external thread screws into the metal plate's internal thread. These additional anatomical reductions were achieved using the dual-lead screw only.

The dual-lead locked plating is the application of additional plate/bone compression utilizing the combined effect of the high stability of the locked plating fixation and the limited compression owing to the dual-lead screw. Compared to conventional screw fixation, the locked dual-lead screw plating can only apply minimal compression because the locking screw head cannot advance any further when its last male thread is locked to the plate's female thread. The findings are new experimentally-proven biomechanical clues for the effective utilization of dual-lead locking screws for surgeons to be able to parametrically adjust plate-bone compression without sacrificing fixation stability or periosteal blood circulation.

Several key findings on the relationship between the lead ratio of the dual-lead locking screw and fixation stability have been identified. First, the dual-lead locking screw elevates the compression between the locking plate and the bone. Second, for the cancellous bone, the elevation in the compression due to the dual-lead thread becomes weaker if the screw body lead is twice larger than the screw head lead. Third, for the cortical/cancellous composite humerus, the elevation in the compression due to the dual-lead thread increases with the screw body lead. Hence, the plate/bone compression varies between the cancellous bone block and the composite humerus.

Thus, the reason the compression trend along the lead ratio differs for the bone block and the humerus is crucial. The bone hardness corresponds with the bone crush intensity. The densities of the tested bone blocks were 10 and 20 PCF with strengths of 2.2 and 8.4 MPa, respectively, whereas the cortical layer of the composite humerus was 100 PCF with 157 MPa strength (Table 1). The cancellous bone block will be crushed more than the composite humerus, and the plate/bone block compression will be released proportionally to the crush. In contrast, the cortical bone layer of the composite humerus directly contacting the plate is strong and will be minimally crushed, resisting the thrusting force without the force release. The deformation caused by the crush can be elastic or plastic. The elastic deformation comes first and continues to resist the applied force and disappears when the force is removed. Conversely, the permanent and unrecoverable plastic deformation that follows elastic deformation cannot resist the applied load. Therefore, we concluded that the plate/humerus compression with strong bone quality withstands higher dual-lead-driven compression.

**Figure 11:**
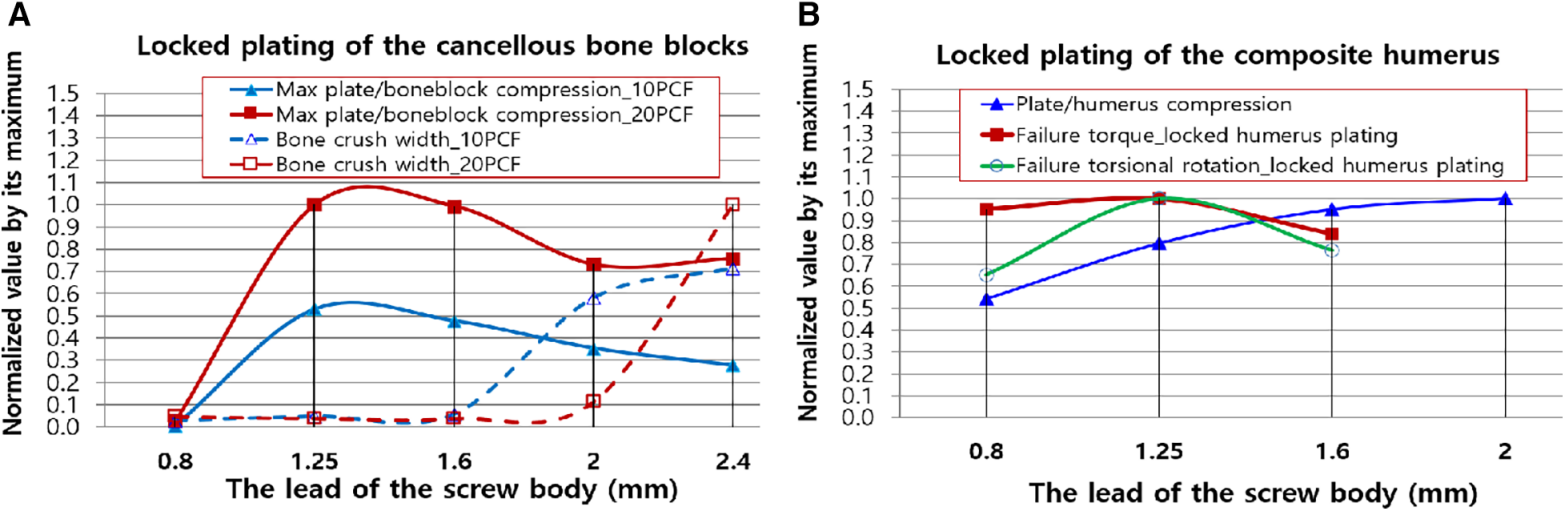
Normalized second-order polynomial curve fits of clinically important results. (**A**) The plate/boneblock compression and bone crush width. (**B**) The plate/humerus compression, torsional failure torque, and rotation of the locked proximal humerus plating. The quantities were normalized using the maximum of each test domain (T1, T2, T3, and T4). The lead of the screw head was 0.8 mm for all the screws.

We think the next three quantities should be regarded clinically more important: the plate/bone compression, the bone crush width, and the torsional fixation stability. For insightful and intuitive investigation of the clinical application of the outcomes, we combined the three quantities and made curve fits of the quantities after normalizing the maximum value of each measurement type (Figure 11).

Figure 11A displays the curve fits of the plate/bone block compression and bone crush width. The compression was high; nevertheless, the bone remained small until the lead of the screw body was 1.6 mm. Thus, when surgeons require additional compression to the locked cancellous bone plating without severely damaging the bone, they can use any locking dual-lead screw whose body's thread lead is <1.6 mm ($R_{\mathrm{lead}}=2$). Figure 11B illustrates the integration of the curve fits of the plate/humerus compression, torsional failure torque, and rotation of the locked proximal humerus plating. Notably, the screws with over $L_{\mathrm{body}}=1.6\;\mathrm{mm}$ ($R_{\mathrm{lead}}=2$) have no advantage in terms of failure torque and maximum torsional deformation compared with the single lead locking screw ($R_{\mathrm{lead}}=1$). However, when surgeons require maximum rotational stability for the locked humerus plating, the dual-lead locking screw of $L_{\mathrm{body}}=1.25\;\mathrm{mm}$ ($R_{\mathrm{lead}}=1.56$) may be considered. Even though its use is not significantly advantageous in terms of failure torque, the fixation using the dual-lead locking screw of $R_{\mathrm{lead}}=1.56$ would withstand approximately 35% more rotational deformation compared to fixation using single-lead locked screw plating.

The natural proximal humerus consists of abundant cancellous bone and relatively thinner cortical bone, and osteoporosis could reduce the cortical thickness of the proximal humerus, thereby increasing its fragility (24). To prevent the increase of fracture comminution in the osteoporotic fracture of the proximal humerus, direct reduction of fracture fragments using forceps or clamps should be avoided, and this pathoanatomical characteristic makes the accurate reduction of fracture difficult. Clinically, stay sutures over the rotator cuffs are frequently used to reduce the fracture fragments and maintain fixation by neutralizing the traction force of rotator cuffs (25); however, in cases of concurrent rotator cuff tears with proximal humerus fractures, the use of stay sutures might be limited. Furthermore, locking plate fixation would provide stronger biomechanical fixation than suture (26). Loss of reduction following locking plate fixation in proximal humerus fractures could increase the reoperation risk (27); therefore, dual-lead locked screw fixation might be helpful to achieve and maintain accurate reduction and ultimately improve the clinical outcomes after osteosynthesis surgery for proximal humerus fractures.

Our study has some limitations. The tested composite humeri were the replica of normal bone density humerus, and the test did not include those with osteoporosis. Osteoporotic proximal humerus fractures may exhibit outcomes similar to those of cancellous bone block tests. Dual-lead locked plating will be a good study to solve the fixation of osteoporotic proximal humerus. Additionally, this study tested only artificial humeri and bone blocks. The cadaveric human bones will provide more realistic results. However, the uniformity in shape and density of the artificial humerus is beneficial, unlike those of a cadaveric humerus. This study investigated the effect of the dual-lead screw without considering variations in anatomical structure or local bone quality. Finally, the maximum or minimum specific values or the trend according to the screw lead ratio may differ for the locked plating for other human body parts because other bone fixations use various screw fixation implants and have different bone quality.

In conclusion, this study demonstrated the plate/bone block interactive mechanics when the dual-lead screws were inserted into locking plates and bones. Additionally, the dual-lead locked plating can apply additional anatomical reduction through limited compression owing to the dual-lead screw of ∅ 3.5 mm diameter. Moreover, any locking dual-lead screw with a body thread lead of <1.6 mm ($R_{\mathrm{lead}}=2$) can be utilized without the risk of bone crush. If pursuing safe fixation stability of the osteoporotic or normal proximal humerus, the dual-lead locking screw with $L_{\mathrm{body}}=1.25\;\mathrm{mm}$ when $L_{\mathrm{head}}=0.8$ is recommended. Our study findings provide surgeons with a biomechanical clue for the effective utilization of dual-lead locking screws when applying additional anatomical reduction during locked plating for proximal humerus fractures.

## Data Availability

The original contributions presented in the study are included in the article/Supplementary Material, further inquiries can be directed to the corresponding authors.
